# Engineered retargeting to overcome systemic delivery challenges in oncolytic adenoviral therapy

**DOI:** 10.1016/j.omton.2025.201005

**Published:** 2025-06-06

**Authors:** Louise Leparc, Hiroaki Wakimoto

**Affiliations:** 1 Department of Neurosurgery, Massachusetts General Hospital, Harvard Medical School, Boston, MA 02114, USA; 2 Laboratory of Nervous System Disorders and Therapy, GIGA Institute, University of Liège, 4000 Liège, Belgium

## Main text

Systemic delivery of oncolytic viruses (OVs) represents a promising, clinically relevant strategy that may enable therapeutic targeting of disseminated tumors and metastases commonly found in advanced cancers. This approach holds the potential to overcome some of the key limitations associated with frequently used intralesional OV injections, such as uneven intratumoral virus spread, inability to target metastatic sites, and inaccessibility for certain tumor locations. However, multiple challenges hinder the clinical utility of systemic OV delivery. First, systemic OV delivery may mediate suboptimal tumor targeting and off-target viral accumulation, which can result in unwanted side effects and organ toxicities. Second, pre-existing neutralizing antibodies (NAbs) against OVs can rapidly eliminate the virus from blood circulation and reduce therapeutic activity. Thus, addressing these obstacles can help optimize systemic OV delivery strategies to improve efficacy and broaden the applicability of oncolytic virotherapy.

**Figure 1:**
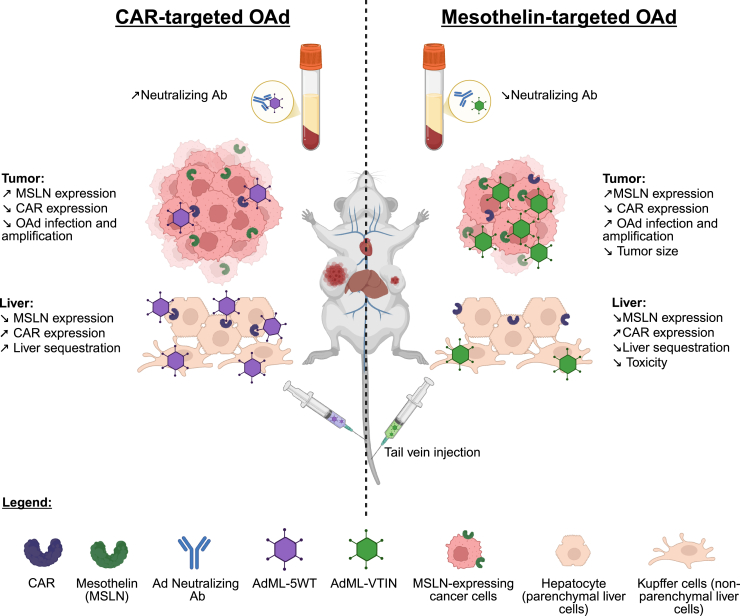
Summary of the advantages of mesothelin-targeted oncolytic adenovirus over CAR-targeted adenovirus following systemic administration Note the differences in impact on neutralizing antibodies, liver sequestration, and anti-tumor efficacy. Created in https://BioRender.com.

In this issue of *Molecular Therapy Oncology*, Sato-Dahlman et al. demonstrated that transductional retargeting of oncolytic adenovirus (OAd) enhanced tumor-specific infection and facilitated anti-tumor efficacy upon systemic delivery while minimizing virus distribution to normal tissues.^1^ The authors’ group previously identified the infectivity-selective, mesothelin-retargeted OAd (AdML-VTIN) through a screening of a high-diversity adenoviral fiber library.^2^ Intravenous administration of AdML-VTIN was safe and, when compared with control OAd without fiber modification (AdML-5WT), enhanced intratumoral viral amplification and subsequent tumor destruction and reduced off-target liver sequestration in mouse models of mesothelin-expressing pancreatic and lung cancer xenografts (Figure 1). Compared with intratumoral injection, intravenous injection resulted in robust virus replication within the tumor, despite lower initial tumor infection. Additionally, AdML-VTIN showed increased resistance to NAbs, supporting its potential as a promising systemic virotherapeutic for advanced malignancies.^1^

Adenovirus infection of cells is mediated by the interaction between the globular knob domain of the viral fiber and the native coxsackievirus and adenovirus receptor (CAR) on the cell surface which is often downregulated in cancer cells. AdML-VTIN is unique in that it has a genetic modification in the AB loop of the fiber knob. Insertion of a “VTINRSA” motif in the CAR-engaging region of the AB loop in AdML-VTIN abrogates interaction with CAR but confers selective binding to mesothelin, redirecting infectivity from CAR to mesothelin.^2^ This feature is distinct from the widely used engineering strategy in OAd development employing genetic modifications in the HI loop of the fiber with the goal to enhance OAd infectivity of cancer cells, rather than fully retargeting OAd infectivity. A well-known example of this approach is the clinical OAd DNX-2401 that carries the Arg-Gly-Asp motif in the HI loop of the fiber, enabling viral binding to αv integrins.

Due to expression of CAR in normal tissues, particularly liver parenchymal cells (e.g., hepatocytes), systemic delivery of CAR-binding OAd is hampered by liver sequestration that reduces the number of viral particles available to reach and infect the tumor, ultimately lowering overall tumor infectivity and compromising anti-tumor efficacy. Off-target OAd accumulation can also contribute to toxicities and adverse inflammatory responses. Disrupting the ability to engage the native CAR receptor and retargeting to tumor-associated mesothelin, intravenously administered AdML-VTIN demonstrated selective binding to and enhanced cytotoxicity against mesothelin-expressing tumor cells and reduced affinity for non-tumoral tissues such as the liver, allowing AdML-VTIN to evade liver sequestration, compared with fiber-non-modified AdML-5WT. However, abrogation of CAR binding did not fully prevent uptake by non-parenchymal Kupffer cells in the liver.

Importantly, modification of the AB loop of the fiber can be applied beyond mesothelin targeting to open up new opportunities to redirect OAd infectivity to cancer-associated cell surface molecules. An OAd with CD133-targeting motif may be tested for systemic delivery to target disseminated CD133^+^ cancer stem cells.^3^ Polyspecific integrin-binding peptides that can bind multiple integrins overexpressed in cancer may represent a candidate to be incorporated into AB loop engineering of OAd, expanding the potential of AB loop-specific fiber modifications for OAd cancer therapy.^4^

NAb-mediated OV neutralization represents another key impediment to systemic OV delivery. Compared with fiber-non-modified AdML-5WT, AdML-VTIN was more resistant to neutralization by human serum. Furthermore, AdML-VTIN and adenovirus type 5 (Ad5)/3 fiber chimera did not induce hemagglutination, unlike AdML-5WT. These observations supported the idea that AB loop modification may render OAd amenable to systemic administration. Various approaches have been proposed to overcome NAb-mediated OV inactivation. Insertion of an albumin-binding domain into the capsid hexon enabled an OAd to associate with serum albumin, thereby providing a protective shield against pre-existing NAbs and enhancing systemic delivery efficacy.^5^ Other strategies rely on cell-mediated delivery, using, for example, mesenchymal stem cells or lymphocytes as OV carriers.^6^^,^^7^ Interestingly, Sato-Dahlman et al. showed that multiple systemic injections of AdML-VTIN led to promising tumor control, with repeated dosing significantly suppressing regrowth in most tumors. These results suggest that AB loop fiber-modified OAd may evade eliciting NAb responses after repeated systemic administration, retaining anti-tumor efficacy. However, this notion will need to be validated in immunocompetent animal models.

Mesothelin is a glycoprotein normally expressed on the surface of mesothelial cells. Notably, mesothelin is overexpressed in various malignancies, including ovarian cancer, pancreatic cancer, lung cancer, mesothelioma, and hematological malignancies, positioning it as a promising target for cancer therapies.^8^ Recently, pre-clinical and clinical studies have focused on targeting mesothelin in solid tumors. RC88 is a mesothelin-targeting antibody-drug conjugate for the treatment of recurrent platinum-resistant ovarian cancer.^9^ Another promising approach to target mesothelin is through CAR-T cells, which enhanced tumor selectivity and minimized off-tumor toxicity in pancreatic cancer.^10^ Other mesothelin-targeting strategies, such as monoclonal antibodies, immunotoxins, and vaccines, are being tested in preclinical and clinical trials.^8^ These studies further validate the potential of mesothelin-targeted therapies in improving cancer treatment outcomes and support the potential of mesothelin-targeting AdML-VTIN for various cancers overexpressing mesothelin. However, it should be noted that mesothelin is not a cancer-specific protein. A certain amount of AdML-VTIN accumulation in the lung was noted following systemic administration, which may be due to mesothelin expression on mesothelial cells lining the pleura.^1^

Several OV platforms have entered clinical evaluation of systemic intravenous delivery for cancer therapy, including RNA viruses (e.g., reovirus and measles virus) as well as DNA viruses (e.g., OAd).^11^ OAd VCN-01, which incorporates a fiber shaft modification, has shown a favorable safety profile and preliminary antitumor activity in patients with advanced pancreatic adenocarcinoma.^12^ Demonstration by Sato-Dahlman et al. that mesothelin-retargeted AdML-VTIN preferentially targeted tumor cells, thereby enhancing therapeutic efficacy and minimizing adverse effects on healthy tissues in the context of systemic therapy, provides a scientific basis supporting the clinical translation of this fiber-modified OAd. However, their investigations were limited to human cell lines and patient-derived xenografts in T lymphocyte-deficient nude mice, the species that is inherently resistant to Ad5. Further preclinical research is needed to assess biodistribution and off-target and dose-limiting toxicities in immunocompetent models, ideally in animals susceptible to Ad5 such as Syrian hamsters.

Ensuring safety is important given the presence of wild-type E1 in AdML-VTIN. Investigations relevant to systemic treatment include NAb and anti-virus immune responses after repeated administrations and cytokine/chemokine release and inflammatory signatures that are linked to adverse effects. Understanding the role of treatment-induced immune cell infiltration and anti-tumor immunity in overall efficacy will also be vital as such immune activities may contribute to the killing of mesothelin-negative tumor cells that may survive after AdML-VTIN-mediated direct elimination of mesothelin-positive tumor cells.

## Acknowledgments

This work was supported by FNRS (reference FC 57667), Wallonie-Bruxelles International (WBI), and Fonds Léon Frédéricq to L.L.

## Declaration of interests

The authors declare no competing interests.

## References

[1] Sato-Dahlman M., Miura Y., Hajeri P., Roach B., Jacobsen K., Yamamoto M. (2025). Systemic therapy with the infectivity-selective oncolytic adenovirus by targeting mesothelin. Mol. Ther. Oncol..

[2] Miura Y., Yamasaki S., Davydova J., Brown E., Aoki K., Vickers S., Yamamoto M. (2013). Infectivity-selective oncolytic adenovirus developed by high-throughput screening of adenovirus-formatted library. Mol. Ther..

[3] Sato-Dahlman M., Miura Y., Huang J.L., Hajeri P., Jacobsen K., Davydova J., Yamamoto M. (2017). CD133-targeted oncolytic adenovirus demonstrates anti-tumor effect in colorectal cancer. Oncotarget.

[4] Miller C.L., Sagiv-Barfi I., Neuhöfer P., Czerwinski D.K., Artandi S.E., Bertozzi C.R., Levy R., Cochran J.R. (2022). Systemic delivery of a targeted synthetic immunostimulant transforms the immune landscape for effective tumor regression. Cell Chem. Biol..

[5] Rojas L.A., Condezo G.N., Moreno R., Fajardo C.A., Arias-Badia M., San Martín C., Alemany R. (2016). Albumin-binding adenoviruses circumvent pre-existing neutralizing antibodies upon systemic delivery. J. Control. Release.

[6] Santos J., Heiniö C., Quixabeira D., Zafar S., Clubb J., Pakola S., Cervera-Carrascon V., Havunen R., Kanerva A., Hemminki A. (2021). Systemic Delivery of Oncolytic Adenovirus to Tumors Using Tumor-Infiltrating Lymphocytes as Carriers. Cells.

[7] Dembinski J.L., Spaeth E.L., Fueyo J., Gomez-Manzano C., Studeny M., Andreeff M., Marini F.C. (2010). Reduction of nontarget infection and systemic toxicity by targeted delivery of conditionally replicating viruses transported in mesenchymal stem cells. Cancer Gene Ther..

[8] Faust J.R., Hamill D., Kolb E.A., Gopalakrishnapillai A., Barwe S.P. (2022). An Immunotherapeutic Target beyond Solid Tumors. Cancers (Basel).

[9] Liu Y., Li G., Yang R., Huang Y., Luo S., Dang Q., Li Q., Huang D., Huang Y., Tang D. (2024). The efficacy and safety of RC88 in patients with ovarian cancer, non-squamous-non-small-cell lung-carcinoma and cervical cancer: Results from a first-in-human phase 1/2 study. J. Clin. Oncol..

[10] Watanabe K., Luo Y., Da T., Guedan S., Ruella M., Scholler J., Keith B., Young R.M., Engels B., Sorsa S. (2018). Pancreatic cancer therapy with combined mesothelin-redirected chimeric antigen receptor T cells and cytokine-armed oncolytic adenoviruses. JCI Insight.

[11] Ban W., Guan J., Huang H., He Z., Sun M., Liu F., Sun J. (2022). Emerging systemic delivery strategies of oncolytic viruses: A key step toward cancer immunotherapy. Nano Res..

[12] Garcia-Carbonero R., Gil Martín M., Alvarez Gallego R., Macarulla Mercade T., Riesco Martinez M., Guillen-Ponce C., Vidal N., Real F., Moreno R., Maliandi V. (2019). Systemic administration of the hyaluronidase-expressing oncolytic adenovirus VCN-01 in patients with advanced or metastatic pancreatic cancer: First-in-human clinical trial. Ann. Oncol..

